# Shoreline Erosion Assessment Modelling for Sohar Region: Measurements, Analysis, and Scenario

**DOI:** 10.1038/s41598-020-61033-y

**Published:** 2020-03-04

**Authors:** E. Abushandi, A. Abualkishik

**Affiliations:** 1 Stream of Civil Engineering, Faculty of Engineering, Sohar University/Sultanate of Oman, Sohar, Oman; 2 Edge Hill University, Ormskirk, Lancashire United Kingdom; 3 Faculty of Computing and IT, Sohar University/Sultanate of Oman, Sohar, Oman

**Keywords:** Hydrology, Ocean sciences

## Abstract

The extended coastlines of Oman have been forced to change in the last few decades because of urbanization development or by natural disasters. Recently, Oman has suffered from a couple of tornados and cyclones, e.g. Cyclone Gonu on June 1, 2007, making the changes even much more dynamic. In order to protect the coastal regions infrastructure, an accurate estimation of shoreline erosion is required. This research paper presents an assessment of shoreline erosion magnitudes using field measurements coupled with Multiple Linear Regressions Models (MLR) to predict future changes. Inverse Distance Weighing and Kriging interpolation methods have been applied in order to visualize shoreline variations from gathered data prospective. The field measurements for the shoreline were taken at 19 different points, the space between the points in a range of 500–700 m approximately. The first field measurements were taken on 19^th^ 20^th^ 21^st^ of June, 2016 while the second field measurements were taken on 14^th^ 15^th^ 16^th^ of November 2016. Pearson correlation shows a strong relationship between the first and the second field trips with an average of 0.83. This significant relationship ensures the applicability of MLRs to project future changes on the shorelines. The results of the MLRs showed severe negative volumetric shoreline erosion with an average of 5.2 m/year with some exceptions at the catchment outlets.

## Introduction

**Figure 1:**
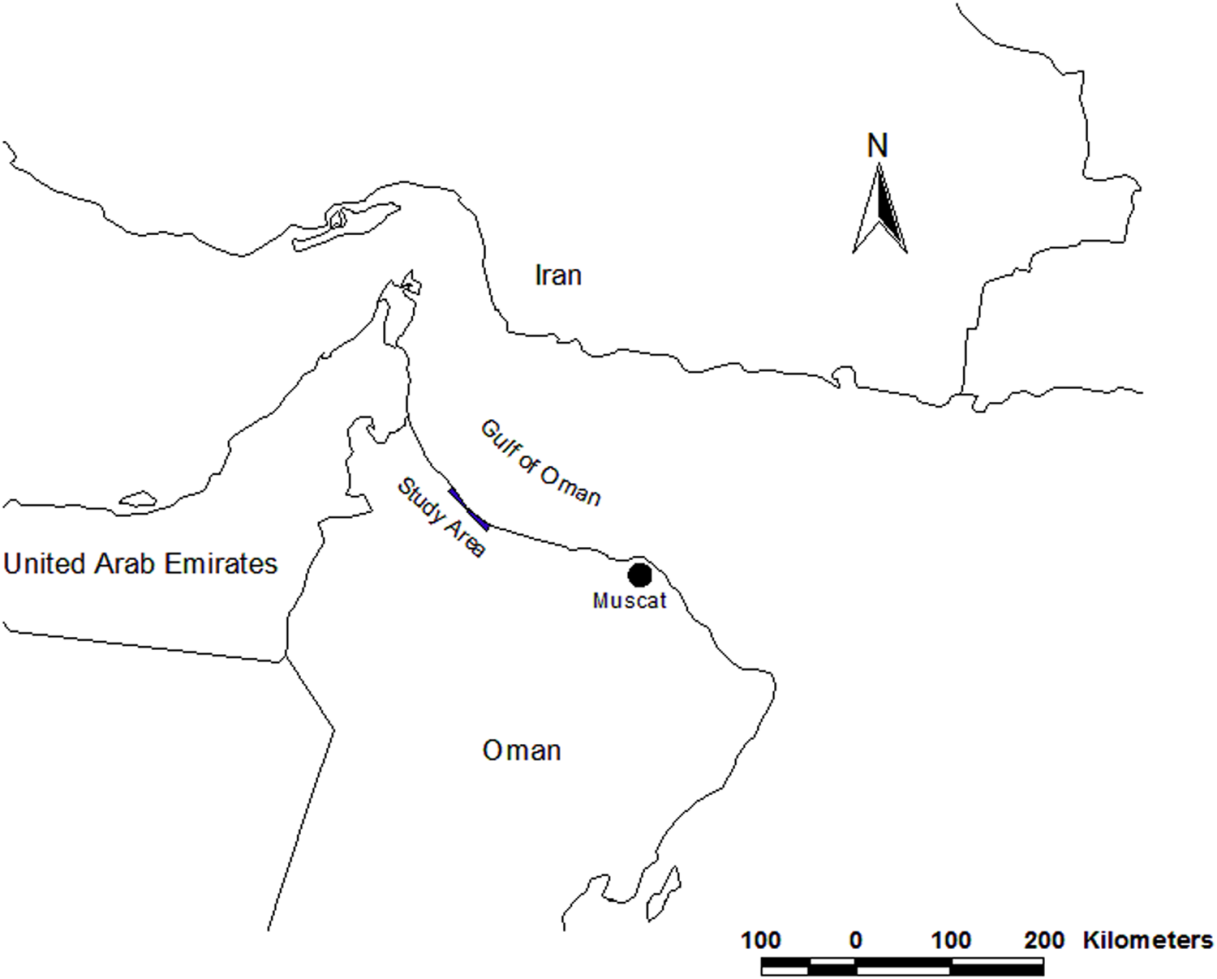
Geographical location of the study area in Oman.

Shoreline erosion is being observed as an important natural process occurring in many places around the world. The shoreline in Oman is overlooking divers three seas; the Arabian Gulf, the Gulf of Oman and the Arabian Sea with around 3,165 km long starting from the Strait of Hormuz in the north to the borders of Yemen in the southwest (Fig. 1).

This line is subjected to continuous erosion and sediment transport. Due to social and economic developments in Oman, shoreline erosion is considered as a serious obstacle affecting the positive development in the country. In order to protect the land and infrastructure, authorized agencies should have a clear plan to manage the shoreline zones effectively. Especially if the sea level rises from unexpected tornados coupled with growing construction rates on the coastlines^1^. However, authorities in Oman have built various types of shoreline protection structures on beaches and corniches in order to limit the impact on urbanized regions^2^ mainly rocks and reinforced concrete which are part of hard stabilization method. They were designed in order to protect the roads and infrastructure from wave’s action.

**Figure 2:**
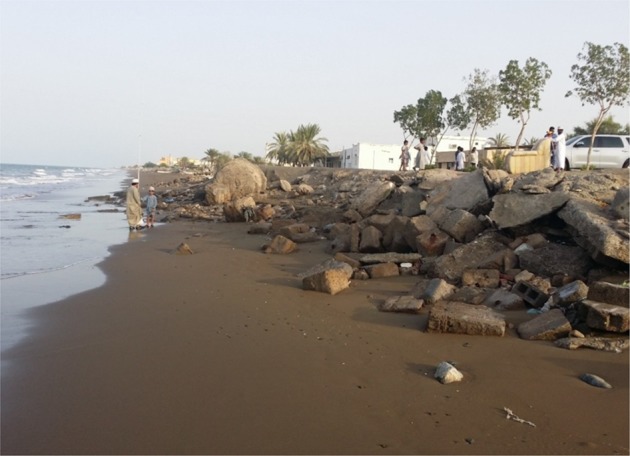
Construction debris used to break the sea waves in the study area.

In addition, there are also some efforts from local individuals to break the sea waves using construction debris (Fig. 2).

Sohar region located in the northern part of Oman is suffering extreme shoreline erosions where owner’s properties and residents are always at risk. Residential communities and commercial buildings are located close to or right on the shorelines^2^, hence, directly affected by any shorelines changes. In addition, more than 55% of the Sultanate’s population lives in Al-Batinah Region and adjacent Muscat Governorate^3^ which are located along the shoreline. Recently, sea level rise represents one key indicator of climate change, consequently, makes the shoreline geomorphological processes even more dynamic. As stated by Church, J. A. *et al*.^4^, it is very likely that in the coming years, sea level change will have a strong regional pattern as a result of global warming, with some places experiencing significant deviations of local and regional sea level change^5^. This, however, will increase the annual mean wave heights and increase the opportunity of shoreline erosion.

There are different assumption and tools used by research specialists to assess shoreline erosion, for example, Rangel-Buitrago *et al*.^6^ and Stanchev *et al*.^7^ Used DSAS 3.2 extension in ArcGIS developed by USGS to identify and analyse the changes in shorelines. An optimal approach is to evaluate the risk of erosion in order to appropriate land use planning. Similarly, De Serio *et al*.^8^ added a new value to this application by rating the key parameters such as physical and socio-economic based on the Analytic Hierarchy Process (AHP). A GIS-based coastal area suitability assessment was carried by Ju *et al*.^9^ to identify the optimal suitability of geo-environmental factors maintaining good environmental condition of the coastal area. They use the Analytic Hierarchy Process (AHP) in order to optimize the results.

In another hand, some researchers have implemented tools that also facilitated analysis of a number of other inherent coastal hazards including ecosystem disruption^10^, gradual inundation, salt water intrusion, and flooding^11^, or even finding the connection between human induced factors (such as dams or reservoirs, dredging, mining, and water extraction etc.) and coastal erosion using remote sensing and GIS techniques^12^. Indeed, there is a growing interest in implementing geospatial satellite-prone data and GIS-based tools to provide a quick assessment of shoreline erosion (e.g. Narra *et al*.^13^, Cellone *et al*.^14^, and Kuenzer *et al*.^15^). Furthermore, geospatial data of shorelines are critical for safe navigation, coastal resource management, coastal environmental protection, and sustainable coastal development and planning^16,17^.

San *et al*.^18^ and Aedla *et al*.^19^ used linear regression methods to predict future shoreline erosion. Basically, the method was applied into satellite images including millions of pixels. The results showed the potential of linear regression method in finding shoreline changes. However, the prediction of future shoreline erosion is influenced by many factors and it’s not always possible to take into account all factors for the entire period. Therefore, the method is subjected to a level of uncertainty of shoreline erosion prediction. In addition, a prediction time of more than 10 years was selected, in that longer period may result an additional prediction error and increased uncertainty.

In fact, shoreline erosion is caused by various factors, including natural processes such as weather parameters, low and high tides which affect the direct wave action, and low topographic area. On the other hand, anthropogenic activities can also cause erosion such as the construction of new engineering projects along the shorelines. Almost 60% of Oman population is living near to the coastal areas and experiencing coastal erosion risks. In Muscat and Al-Batinah where the population density is higher than other regions the rate of erosion is considered as a serious hazard. The most common projects may accelerate shoreline erosion include new human settlement, industry, fishing, transportation. Particularly, Badenhorst^20^ considered Al-Batinah region to be a state of widespread erosion with possible causes of instable shoreline, sea level rise coupled with industrial harbour and dams construction. Moreover, flash floods in such arid regions cause a significant disturbance at the Wadis coastal outlets.

Although it’s strenuous to differentiate the impact of climate on shoreline erosion, there are some research efforts to recognize this impact such as Cabral *et al*.^21^. They use an open source model called Invest where land geomorphology, Habitats wind and wave exposures are the main parameters to run the model. Anthropogenic impacts on shoreline erosion have been also discussed by many researchers (e.g. Al-Madany *et al*.^22^, Short and Wyllie-Echeverria^23^, Syvitski *et al*.^24^, and Neil *et al*.^25^, and Kirwan *et al*.^26^).

The overall goal of this research is to develop a regional shoreline erosion assessment model which can be applied for other coastal regions. Based on a conventional survey, the assessment includes actual measurements as well as future erosion projections.

## Study Area

**Figure 3:**
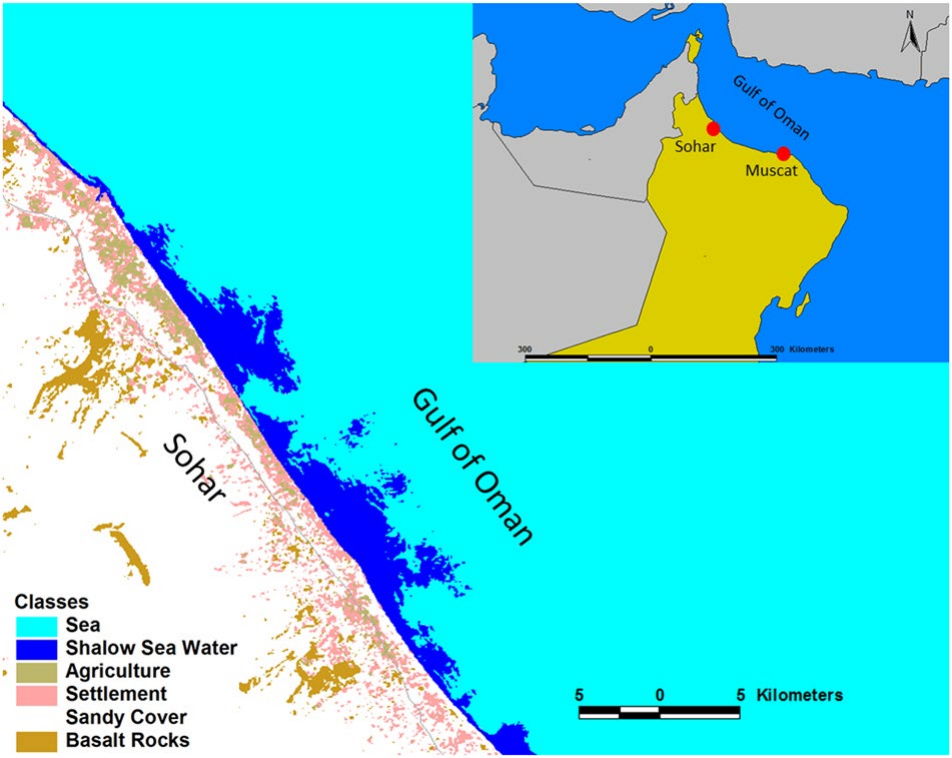
Location map of Sohar study area along with the Gulf of Oman coast. The maps were generated using ArcMap [10.1] (http://www.esri.com/).

The area considered for this study is Sohar northern part of Oman (Fig. 3). The length of considered sandy shoreline is approximately 18 km of Sohar area and characterized by a gently flat morphology. According to one principle of classification, the shoreline of Sohar is considered as a submergent coastline where the sea level has risen due to global sea level changes.

From the hydrogeological point of view, the catchment area is arid characterized by a heavy flash flood in winter season. The use of groundwater aquifers is generally limited because of the risk of saltwater upcoming.

## Instrumentation and Methodology

**Figure 4:**
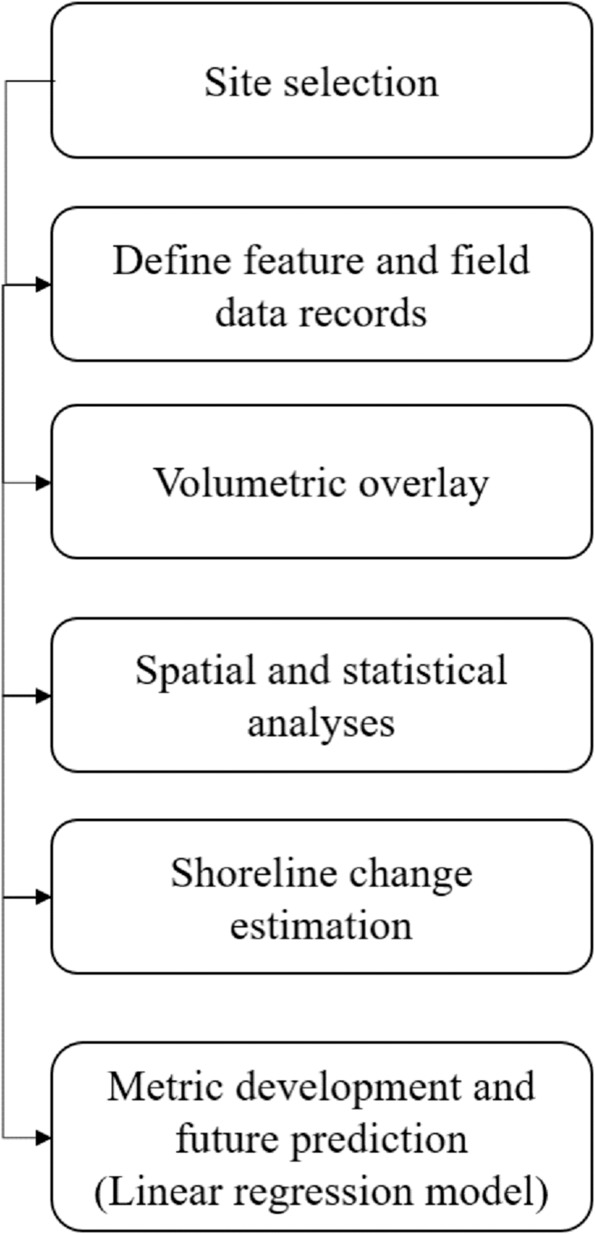
Flowchart outlines the methods used in this study.

Capturing the spatial heterogeneity of shoreline erosion in the region is crucial for volumetric erosion assessments. Therefore, the selected procedure should be able to explain the relationship and feedback mechanisms between potential surface dynamics and hydro-meteorological variables (Fig. 4). The procedure selection is based upon two main principals: Availability and quality of data, especially field parameters.

**Figure 5:**
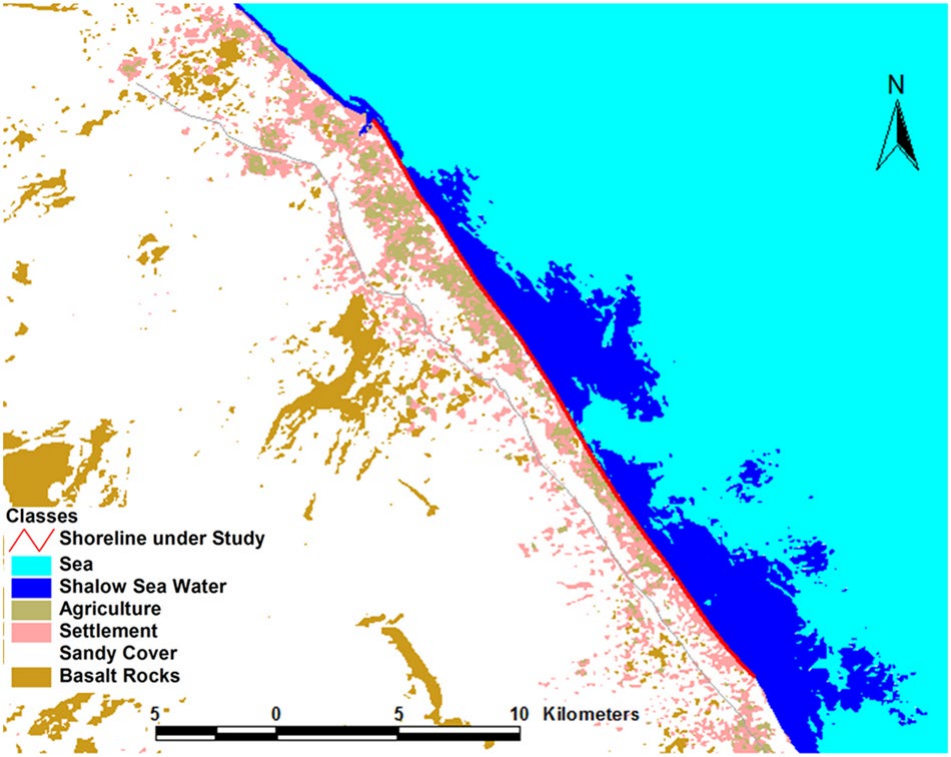
Measurements area within Sohar shoreline.

To ensure the seasonality of measurements, the data of this research was based on two conventional surveys at five months’ period providing precise details on horizontal and vertical small changes. The field measurements of the shoreline were taken at 19 different points, the space between the points is 500 m approximately. The first field measurements were taken on following dates 19^th^,20^th^, and 21^st^ of June, 2016 and the second field measurements were taken on date 14^th^, 15^th^, and16^th^ of November 2016, taking into account the moon calendar instead of Georgian calendar. The reason of considering the moon calendar is to have the same effect of moon gravity on the study case. Discretization of the Sohar shoreline into a geometrical number of grids gives a chance to accurately assess erosion and project future scenarios with clear descriptions of shoreline dynamic. Each point of measurement represents elevation values at the two conventional surveys. The study area for shoreline area is approximately 18 km of Sohar shoreline line (Fig. 5).

**Figure 6:**
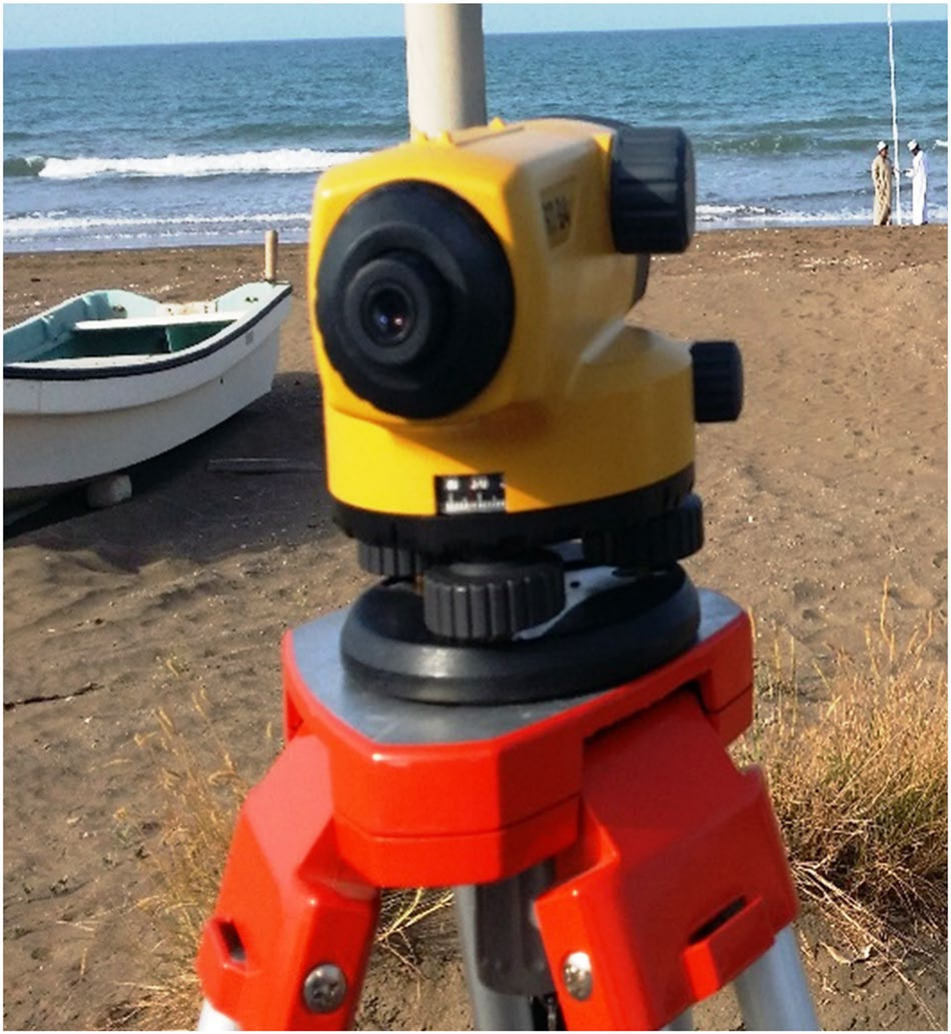
Leica Automatic Level used during the field measurements.

Leica Automatic Level was used to determine the difference height between points and heights of points above a datum surface (Fig. 6). In addition those parameters have also measured:

i. Measure dry water line width and shoreline elevation at a point
ii. Monitor seasonal short- term shoreline geomorphologic changes.

The differential GPS device has been used to orient each point of measurement during the visits. All points have been recorded in order to benchmark them with further field measurements. Benchmarking the points from different field measurements will provide information on the eroded volumes and vertical stability in its immediate vicinity. These defined points are also important to understand the spatial relationship and feedback mechanisms of erosion dynamic processes. In addition, interpolation of those points will provide information on spatial variations of the shoreline. Inverse distance weighted (IDW) and Kriging interpolation methods were applied in order to create shoreline maps of gathered data. The IDW interpolation explicitly depends on the assumption that closer objects that are more alike than those are farther apart (Grain^27^).

IDW is given by:

$${Z}_{j}={k}_{j}\mathop{\sum }\limits_{i=1}^{n}\,\frac{1}{{d}_{ij}}{Z}_{i}$$

$${k}_{j}=\mathop{\sum }\limits_{i=1}^{n}\,\frac{1}{{d}_{ij}}$$

Where the value *k*_*j*_ in this expression is an adjustment to ensure that the weights add up to 1. *Z*_*i*_ is the value of knwon point. *d*_*ij*_ is distance to known point. *Z*_*j*_ is the unknown point. *n* is a user select exponent.

In addition, Kriging is similar to IDW in the way that the weights of the surrounding measured values can be used to derive unmeasured values based on the location. However, there are different modified formulas of Kriging interpolation seeking high accuracy. The general formula for kriging method is as following:

$$\hat{Z}({S}_{o})=\mathop{\sum }\limits_{i=1}^{N}\,{\lambda }_{i}Z({S}_{i})$$

**Figure 7:**
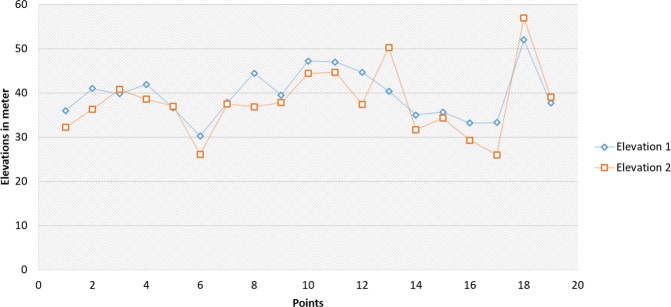
3D Surface maps for the first observations and the second observations (IDW and Kriging interpolation methods).

Where Z (*S*_*i*_) is the measured value at the location *i*, while *i* itself is an unknown weight for the measured value at the ith location; ${S}_{o}$ is the prediction location; *N* is the number of measured values. Golden Software Surfer was used to model the surface and create grid-based maps and 3D surface mapping (Fig. 7).

Perform a sensitivity analysis which provides a ranking of the model inputs based on their relative contributions to model output variability and uncertainty.

Pearson correlation was conducted to determine how strong that relationship is between the first and the second measurements. The correlation formula as following:

$${\rm{r}}=\frac{{\rm{N}}\sum {\rm{xy}}-(\sum {\rm{x}})(\sum {\rm{y}})}{\sqrt{[{\rm{N}}\sum {{\rm{x}}}^{2}-{(\sum {\rm{x}})}^{2}]}[{\rm{N}}\sum {y}^{2}-{(\sum y)}^{2}]}$$

r: Pearson Coefficient

N: is number of pairs of scores

x: is the sum of the first field measurements elevation points

y: sum of the second measurements elevation points

The Pearson correlation coefficient (r) value ranges between −1.00 and +1.00. Achieving a value closer to −1.00 and +1.00 indicates that shoreline data points are aligned with the best fit of linear relationship, while closer to 0 means the variation is great between the two variables. Consequently, linear regression was conducted in order to find future changes. The linear model will be able to predict future volumetric losses.

In addition, the results of the models will be compared in respect to the field measurements and interpolation methods. There are three main software packages to be used for this research: ArcGIS, Golden Software Surfer, and Statistical Package for the Social Sciences (SPSS). ArcGIS was used to create maps and grid files, as well as, analyse the geospatial shoreline data; and compiling Remote Sensing with Field Surveys data. Grid files are required to produce grid based maps including contour and raster maps. Golden Software Surfer has been extensively used for shoreline interpolation, and volumetric surface analysis. Furthermore, SPSS is performing the statistical part of the research, applying Linear Model and time frequencies analyses for various parameters.

## Results and Discussion

**Figure 8:**
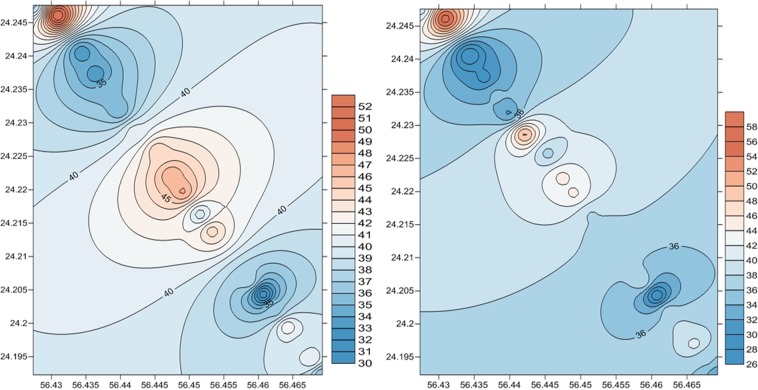
Elevation measurements for the two conventional surveys.

The results provide a clear overview of the current coastal erosion risk at the Gulf of Oman. The study also presents a key methodology in data gathering needed to be addressed in order to develop more effective long-term shoreline management. The mean values of shoreline elevations for the first and the second field measurements were 39.65 m and 37.37 m respectively. While the standard deviation values were 5.57 for the first visit and 7.66 for the second visit. However, the temporal data comparison between the two conventional surveys shows that there is a significant change in shoreline elevations at the same point within the five months period (Fig. 8). Most of the elevation records from the second survey are lower than the first survey except two points where the records were higher indicating the loose in shoreline volume. A lagoon and an outlet from an extended catchment were the reasons for having those higher two values.

**Figure 9:**
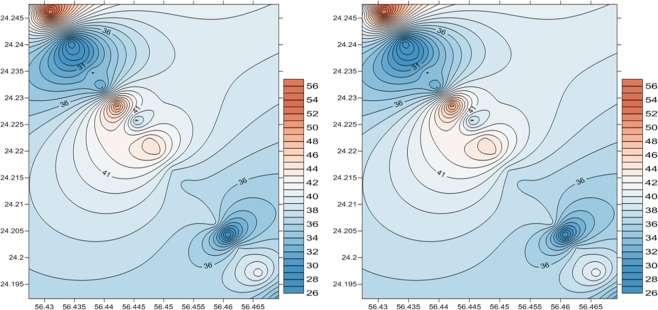
Contour Maps using IDW method for the first elevation (left) and the second elevation (right).

**Figure 10:**
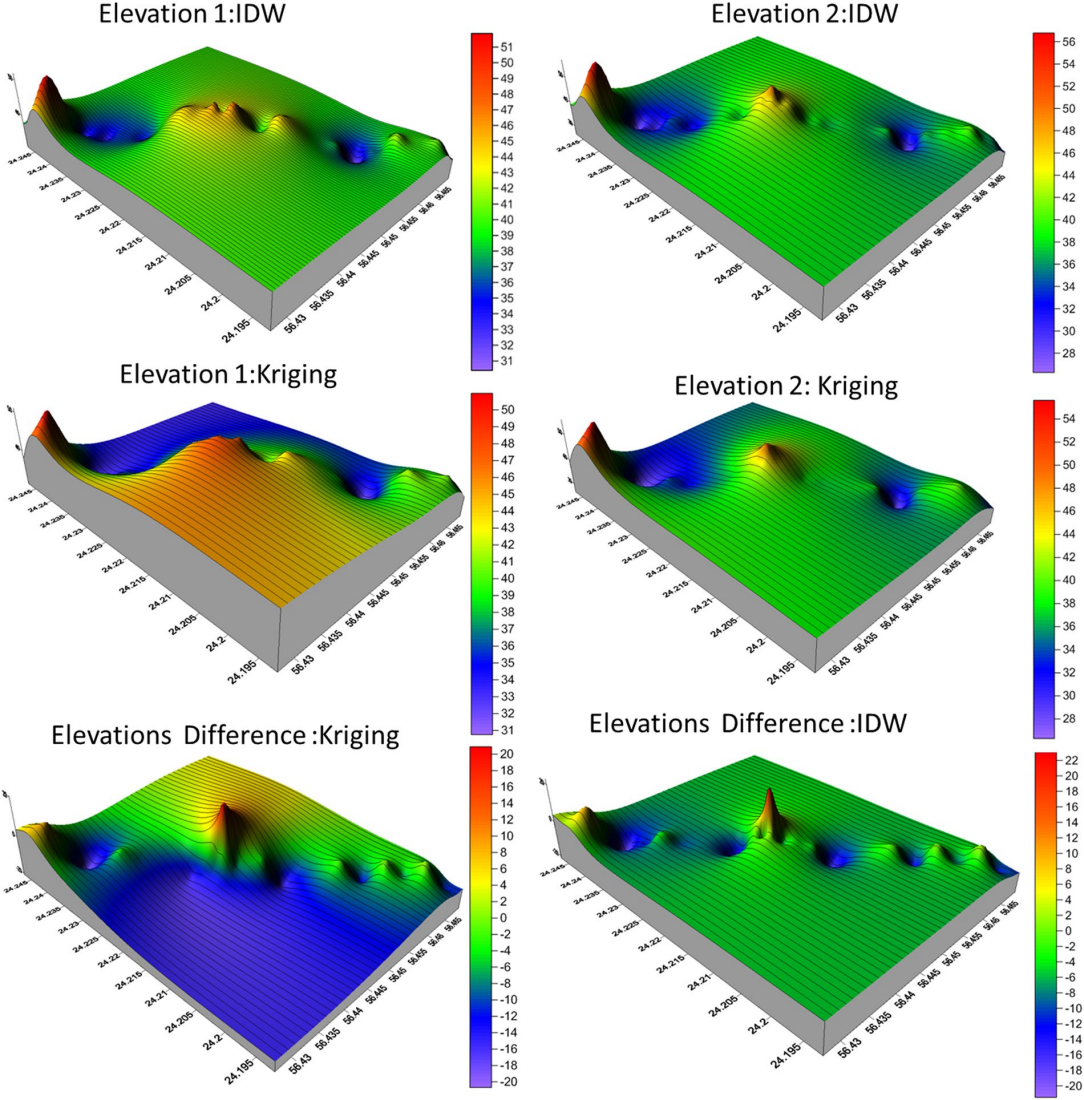
Contour Maps using Kriging method for the first visit elevation (left) and the second elevation (right).

Contour Maps for both readings elevation 1, elevation 2 and reading elevation Reading difference were created using IDW and Kriging Methods (Figs. 9 and 10).

This step is performed by applying a specific tool developed using the Golden Software environment. Although that there is a generic difference between IDW and Kriging methods, the results show that a higher erosion gradient particularly lies over north parts of the study area. This short-term change may indicate natural and human impacts along the shoreline.

3D Surface Maps were also produced for the two measurements. Since the IDW and Kriging functions based on ground observations, an iteration algorithm was employed. However, the 3D maps provide a better spatial understanding of the shoreline changes (Fig. 7).

Pearson correlation shows a strong relationship between the first and the second field measurements with a value of 0.83. In another words, shoreline erosion volume may have an error of 17% range (Table 1) which is reasonably acceptable.

**Table 1:** Pearson correlation between the first and the second field measurements.

		1^st^	2^nd^
1^st^	Pearson Correlation	1	0.830
2^nd^	Pearson Correlation	0.830	1
	N	19	19

Linear regression analysis concluded that the dynamic of shoreline changes is linear. For regression analysis the input data were two variables, independent value was the reading from first field measurements while dependent values were the reading from the second field measurements. The coefficients of regression variables entered to the SPSS is shown in Table 2.

**Table 2:** MLR model coefficients.

Model		Unstandardized Coefficients		Standardized Coefficients	T	Sig.
		B	Std. Error	Beta		
1	(Constant)	−7.886	7.448		−1.059	0.305
	1^st^	1.141	0.186	0.830	6.132	0.000

a-Dependent Variable: 2^nd^.

The resulting linear equation to estimate shoreline volume losses as following:

$${{\boldsymbol{f}}}_{{\boldsymbol{n}}}=1.141\times {{\boldsymbol{f}}}_{{\boldsymbol{n}}-1}-7.886$$

Where:

${{\boldsymbol{f}}}_{{\boldsymbol{n}}}\,:\,$Modelled shoreline losses in meters for the next 5 months’ period

${{\boldsymbol{f}}}_{{\boldsymbol{n}}-1}\,:$ Previous shoreline measurements in meters

The results of Pearson correlation between modeled and elevation values obtained by linear regression, IDW and Kriging interpolation methods showed that Kriging interpolation gives a slight better correlation than IDW with correlation coefficients of 0.982 and 0.971 respectively. The reason of this difference is because IDW works best with evenly disturbed points while Kriging includes random components where exact location of the point is not known by the function. In addition, IDW is greatly influenced by the outlier which is not the case for Kriging method.

**Figure 11:**
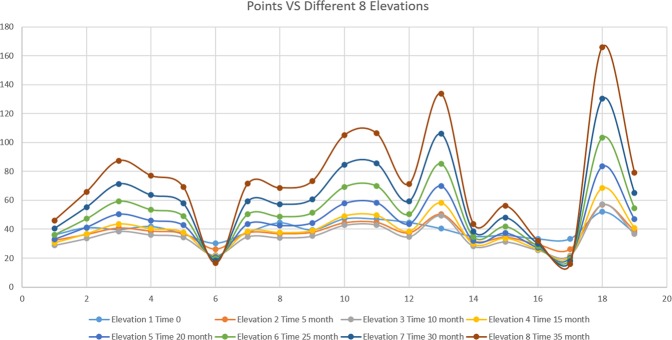
Present and expected future shoreline losses for the next 35 months based on the linear regression model.

The future shoreline volume losses have been projected for the next 30 months at the same points in addition to the first two measurements. Based on the linear regression analysis the point number 18 has the highest erosion among all measurement points which reflects a higher risk, while point number 6 has the lowest erosion volume. Although the area under study is small; the results indicate a high fluctuation rate between the points. The result of this projection has to be seriously considered as the losses in some cases are very high (Fig. 11).

Indeed, the acquisition time of these points is considered critical because of tidal activities and climatic variables. Therefore, it is important to consider the seasonal and annual changes. These distortions may lead to misinterpretation. In order to estimate shoreline change impact on the land cover sensitivity, analysis of land cover type in the study area is extremely important.

This might give the reader a false impression that other forces such as human activities are not important in changing shoreline. Considerations of all forces that might change shoreline and create unstable conditions are important to be addressed. In fact, often the researchers found the change of shoreline at a higher level of the landscape impact. However, measuring historical shoreline change and cliff retreat is also an essential aspect of understanding the long term geomorphic evolution of any coastal system (Stanchev *et al*.^7^). Furthermore, there are some researchers tried to overcome the lack of long term *in situ* measurements using remote sensing images, which have the advantage of providing long-term spatial multi-acquisition image archives (e.g. Yin, P. *et al*.^28^, Misra and Balaji^12^, and García-Rubio *et al*.^29^). In addition, the period of data measurements and intervals are also critical for predicting future changes.

While this research provides the first analysis of the shoreline changes of Omani coast, indicating a seriousness of the shoreline loss, it also offers a methodology that can be used to investigate other coastal regions combining field survey and numerical modeling. Finally, the present research will enable the decision makers to identify the zones with a higher risk and find better solutions to the existing coast problems.

## Conclusion

The results of the analysis show occurrence of severe negative volumetric shoreline erosion with an average of −5.2 m/year with some exceptions at the catchment outlets. In general, the results showed unstable shoreline even for a short line. The shoreline erosion measurements may include effectively actual measures, use of new technologies, as well as scenarios of future projections. Manage the shoreline area effectively especially if the sea level rise from unexpected factors coupled with growing construction rates on the coastal regions is highly important. Detection of shoreline area changes were quantified by analysis of data by GIS base software. The two field measurement had taken with in a period of five months. To provide highly accurate analyses the number of measured points must be greater as well as the number of visits. Weather parameters and sea depths can be considered for future research as important parameters in the multiple linear models. Despite this preliminary effort in quantifying shoreline change, the relationship between coastal erosion and wave height and direction is still poorly understood for the entire country.
